# Effectiveness of a Lifestyle Modification Program Delivered under Real-World Conditions in a Rural Setting

**DOI:** 10.3390/nu13114040

**Published:** 2021-11-12

**Authors:** Cally Jennings, Elsie Patterson, Rachel G. Curtis, Anna Mazzacano, Carol A. Maher

**Affiliations:** 1 Sonder, Edinburgh North, SA 5113, Australia; epatterson@sonder.net.au (E.P.); amazzacano@sonder.net.au (A.M.); 2 Alliance for Research in Exercise, Nutrition and Activity, Allied Health and Human Performance, University of South Australia, Adelaide, SA 5001, Australia; rachel.curtis@unisa.edu.au (R.G.C.); carol.maher@unisa.edu.au (C.A.M.)

**Keywords:** physical activity, nutrition, health program, lifestyle, weight management, prevention, service evaluation, health service

## Abstract

Whilst there is considerable evidence to support the efficacy of physical activity and dietary interventions in disease and death prevention, translation of knowledge into practice remains inadequate. We aimed to examine the uptake, retention, acceptability and effectiveness on physical activity, physical function, sitting time, diet and health outcomes of a Healthy Eating Activity and Lifestyle program (HEAL^TM^) delivered under real-world conditions. The program was delivered to 430 adults living across rural South Australia. Participants of the program attended weekly 2 h healthy lifestyle education and exercise group-based sessions for 8 weeks. A total of 47 programs were delivered in over 15 communities. In total, 548 referrals were received, resulting in 430 participants receiving the intervention (78% uptake). At baseline, 74.6% of participants were female, the mean age of participants was 53.7 years and 11.1% of participants identified as Aboriginal and/or Torres Strait Islander. Follow-up assessments were obtained for 265 participants. Significant improvements were observed for walking, planned physical activity, incidental physical activity, total physical activity, 30 s chair stand, 30 s arm curl, 6 min walk, fruit consumption and vegetable consumption, sitting time and diastolic blood pressure. Positive satisfaction and favourable feedback were reported. The healthy lifestyle program achieved excellent real-world uptake and effectiveness, reasonable intervention attendance and strong program acceptability amongst rural and vulnerable communities.

## 1. Introduction

Poor diet and insufficient physical activity are leading modifiable causes of death and disease [1]. They increase the risk of developing chronic health conditions, such as cardiovascular disease (CVD), type 2 diabetes, obesity, cancers, depression and anxiety, leading to premature death and reduced quality of life, and massive economic and healthcare burden [2]. In Australia, over ninety percent of adults do not consume the recommended daily intakes of vegetables and fruit [3], and two-thirds do not meet guidelines for 30 min of physical activity per day [4].

International evidence consistently shows that physical inactivity and poor dietary patterns disproportionately affect people residing in rural areas, and those who are socioeconomically disadvantaged [5,6,7,8]. People living in rural areas experience poorer health outcomes in comparison to those living in metropolitan areas due to skills shortages and high turnover of healthcare staff, reduced access to and use of preventative health services, as well as disparities in employment, income and education [9]. In addition, lifestyle behaviours and health risks vary based on ethnicity. In particular, Indigenous people tend to have poorer lifestyles and experience worse health outcomes than non-first nation counterparts including an increased risk of chronic disease such as diabetes and shortened life expectancy [10,11,12]. Clearly, there is an urgent need for effective programs to better support people residing in rural areas and high-risk groups to adopt healthier lifestyles.

There is a great deal of evidence supporting the efficacy of physical activity and dietary interventions among adults in the scientific literature [13,14,15]. Evidence has supported improvements in total physical activity, cardiorespiratory fitness, reduced caloric intake and consumption of saturated fat, and an increased intake of fruit and vegetables [15,16]. This includes the delivery of interventions across a variety of modalities (e.g., individual, group based, telephone, print, web-based), settings (e.g., communities, workplaces and healthcare settings) and target groups [16,17]. Yet, such programs are typically evaluated under tightly controlled conditions, such as through randomised controlled trials (RCTs), which limits their external validity, and fail to consider the complexities surrounding delivery and adoption in practice within “real-world” settings [18]. These complexities can include differences in uptake among the public, competing demands on staff, organisational processes and priorities, and resourcing considerations [18,19]. Thus, a gap remains in the translation and implementation of research into practice, and the generalisability of these programs and results to real-world conditions is unclear [18,20,21].

Real-world trials are needed to help close this gap. Two evidence-based programs that have been evaluated under real-world conditions and reported in the peer-reviewed literature are studies based on the US Diabetes Prevention Program and the Australian Healthy Eating Activity and Lifestyle (HEAL^TM^) program. A systematic review of 28 real-world studies of lifestyle programs modelled on the Diabetes Prevention Program, on average, led to 4 percent weight loss [22]. Not surprisingly, the Diabetes Prevention Program-based programs have been heavily targeted at people with pre-diabetes (25 out of 28 studies), so the effectiveness for people with other chronic diseases and risk factors is unclear.

In Australia, the HEAL^TM^ program has been developed as an evidence-based group-delivered healthy lifestyle intervention. A real-world pre–post evaluation among participants (*n* = 2827) across 67 local government areas suggested the program leads to measurable improvements in physical activity, sitting time, fruit and vegetable consumption, anthropometry, and physical function [23]. However, results were only reported in brief; an overly simplistic statistical approach was used (*t*-tests) and did not consider or account for differences in program effectiveness based on sex, age, ethnicity, and it is also unclear which settings the program was tested in (e.g., rural or metropolitan).

This study helps to address these gaps in the literature, offering an analysis of the HEAL^TM^ program delivered under real-world conditions in rural and Indigenous settings such as Aboriginal Controlled Community Health Organisations (ACCHOs). In particular, we aimed to: (1) describe the program’s uptake, retention and engagement; (2) examine the effectiveness of the program for improving physical activity, sedentary behaviour, diet, health outcomes and physical function; and (3) describe participants’ and health professionals’ views on program acceptability and satisfaction. Understanding how healthy lifestyle programs work in real-world settings and with rural and vulnerable communities is essential to address chronic disease risk and management.

## 2. Materials and Methods

### 2.1. Design

This study uses a mixed quantitative and qualitative methods, pre–post design comprising of the delivery of the HEAL^TM^ program—a group-focused lifestyle program implemented in a ‘real world’ primary health care setting. The HEAL^TM^ program is an evidence-based program, developed by South Western Sydney Primary Health Network and supported by Exercise & Sport Science Australia (ESSA) [23]. Sonder was funded by the Country South Australia Primary Health Network to implement the HEAL^TM^ program across rural South Australia. A project officer employed by Sonder was responsible for the implementation of the program. This included undertaking regular promotion and engagement activities with local General Practitioners (GPs) and practice nurses to encourage and support referrals into the program. The project officer recruited local allied health professionals who were subsequently trained and certified to deliver the HEAL^TM^ program as facilitators through ESSA. Local facilitators also engaged with local GPs to support referrals and local community engagement. Intervention and data collection took place between 1 July 2018 and 30 September 2019. Participants provided written consent to partake in the program and for their data to be used for program evaluation purposes. This retrospective analysis of quality assurance data was deemed to be exempt from ethics approval by the University of South Australia’s Human Research Ethics Committee (application no. 204196).

### 2.2. Participants and Procedure

To be eligible, participants were required to be referred by a GP or Nurse Practitioner located in one of the following South Australia regions: Gawler, Barossa, Lower North, Mid North, Yorke Peninsula, Far West, Flinders and Port Augusta, Lower Eyre and Upper Eyre. Participants were eligible for referral if they met one of the following criteria: CVD or 2+ CVD risk factors, were diagnosed with Type 2 diabetes, were pre-diabetic, or had a body mass index (BMI) ≥30. Further eligibility criteria assessed upon receipt of the referral included aged 18 years or over and completion of pre-exercise screening [24] with appropriate medical practitioner approval, if required.

Participant referrals were sent from the general practice to Sonder, where they were processed and allocated to a HEAL^TM^ facilitator located in their region of residence. Facilitators contacted participants via phone to enrol them into the next available program. Participant anthropometry, blood pressure, and physical function assessments were conducted by facilitators in-person prior to the participants’ commencement in the program and immediately following program completion (8 weeks). Participants completed a paper survey assessing their physical activity, sitting time, and diet at baseline and 8 weeks and were invited to complete an online or paper-based satisfaction survey following the completion of the program. 

Stakeholders, including program facilitators, referrers and practice/service managers, were invited (*n* = 30) to complete an online survey to provide feedback about the program in June 2019.

### 2.3. Intervention

The HEAL^TM^ program is an 8-week, group-based lifestyle program that is targeted towards adults with or at risk of developing chronic diseases. Allied health professionals were trained to deliver the HEAL^TM^ program as a facilitator through a 1-day training course run through ESSA. The intervention is guided by the Transtheoretical Model and Stages of Change theory [4] and includes a focus on self-efficacy to support a self-management approach to encourage autonomy and goal-setting for sustained behaviour change [5]. The HEAL^TM^ program included a weekly 2 h group-based session over an 8-week period. Programs varied in relation to both time of day and day of delivery. Group sessions were delivered face-to-face and included 1 h of supervised exercise and 1 h of lifestyle education focused on promoting physical activity and healthy eating through a modified Mediterranean diet approach [6]. Supervised exercise sessions varied weekly and involved low- to moderate-intensity aerobic and resistance activities, which are modified to suit the needs and interests of the participant group. The most common sessions comprised of gym-based, circuit-style exercises where participants followed a prescribed workout, modified according to their fitness level and needs. This commonly included a 10 min warm-up, followed by the main exercise session (free weights, weight machines and/or cardio) for 30–40 min, with a 10-minute warm down. Facilitators monitored and assessed exercise intensity using the perceived exertion scale provided in HEAL^TM^ program materials and/or clinical judgement. As part of the program, materials consisting of education slides, resources and home-based activities including exercises were provided to all participants. Participants also received one-on-one health consultations to assess current fitness, plan an appropriate exercise program, and measure and monitor ongoing progress during and following the program. A comprehensive overview of the intervention has been previously described [2].

### 2.4. Measures

Demographic data were collected at the referral stage, which included date of birth, gender, Aboriginal and Torres Strait Islander status, and postcode. Remoteness was derived from postcode using the Accessibility and Remoteness Index of Australia (ARIA+) [25]. Socioeconomic status categories were derived from postcode using the 2016 Socio-Economic Indexes for Australia (SEIFA) index of socioeconomic disadvantage national decile ranking [26].

Physical activity questions were based on the Active Australia Survey [27] and included weekly minutes spent: walking for more than 10 min; completing other physical activity (not walking); or gardening or household chores that made participants breathe hard. The Active Australia Survey has demonstrated acceptable reliability (*r_s_* = 0.56–0.64) and validity (*r_s_* = 0.52) [28,29] compared with accelerometer data. In addition to summarising the modes of physical activity separately, the three physical activity variables were combined to calculate total weekly physical activity time. 

Sitting time was captured by a single item measuring the average number of hours per day in the previous week spent in sedentary activities [30].

Daily fruit and vegetable consumption was assessed by two questions: one which asked the number of servings of fruit per day and another which asked the number of servings of vegetables per day. Each question provided examples and serving size equivalents. These questions were taken from the valid and reliable Fat and Fibre Barometer questionnaire [31].

Height (cm), weight (kg), waist circumference (cm), hip circumference (cm), and blood pressure (mm/Hg) were measured objectively. Stadiometers were used to measure height; digital scales were used to measure weight; and waist/hip circumference were measured using tape measures. Blood pressure was measured once with the participant seated using a clinical grade sphygmomanometer. The brand and model of instruments varied based on facilitators’ access to equipment; however, participants’ baseline and follow-up assessments were collected using the same instrument. Physical function measures included the 6 min walk test, 30 s arm curl, and 30 s chair rise [32,33].

Weekly program attendance was recorded by the facilitator. The participant program satisfaction survey was delivered in paper form and consisted of 13 items with a mix of Likert scale and open-ended questions [23]. The stakeholder satisfaction survey was delivered via SurveyMonkey and consisted of 15 items with a mix of Likert scale and open-ended questions. 

### 2.5. Statistical Analysis

Baseline participants’ characteristics were analysed descriptively. Differences between those who accepted and did not accept their referral, and differences between those who completed and did not complete the 8-week follow-up assessments, were assessed using one-way analysis of variance and chi-square with post hoc Bonferroni-corrected *z*-test pairwise comparisons. Analyses were conducted in SPSS 25 (IBM Corp., Armonk, NY, USA).

To account for repeated measures and the hierarchical data structure (participants nested within program sites), linear mixed models with restricted maximum likelihood (REML) estimation were used to examine the effectiveness of the program. Random effects were specified to account for the hierarchical structure of the data and time was specified as a fixed effect. Consistent with the principle of intention-to-treat [34], REML allows all available data to contribute to model parameters. Analyses were conducted in Stata15.1 (StataCorp., College Station, TX, USA). An alpha of 0.05 was used to denote statistical significance.

Descriptive statistics for program satisfaction and stakeholder feedback was reflected as percentages of participants responding across the Likert scale items. Open-ended questions were analysed thematically using Microsoft Word.

## 3. Results

### 3.1. Uptake and Retention

Forty-seven programs were delivered across more than 15 communities, including the regions of Lower/Mid North, Lower Eyre, Yorke Peninsula, Gawler/Barossa, ACCHOs and Remote/Royal Flying Doctor communities.

**Figure 1:**
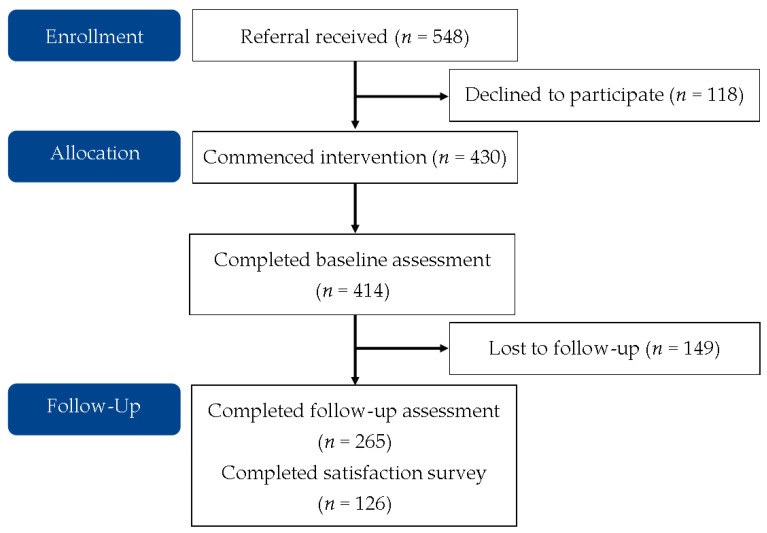
Flow of participants through program.

Figure 1 shows participant flow through the program. A total of 548 referrals were received with 129 GPs or nurses referring at least one person into the program. A total of 430 people accepted the referral and received the intervention.

A comparison was undertaken of those who accepted vs. those who declined the referral (Table S1). A greater proportion of females (81.0%) compared to males (71.1%) accepted the referral (*p* = 0.02). Additionally, a greater proportion of those without a healthcare card (90.9%) compared to those with a healthcare card (81.9%) accepted the referral (*p* = 0.01). Acceptance also differed based on remoteness (*p* = 0.01); rates of acceptance in major cities (57.9%) was similar to inner regional (81.6%), outer regional (73.8%), and remote SA (81.0%), but lower than very remote SA (100.0%). There were no significant differences for age, socioeconomic status, or Aboriginal and/or Torres Strait Islander status for uptake in referrals.

Four hundred and fourteen participants completed baseline assessments. Table 1 shows participant characteristics at baseline.

At baseline, 74.6% of the participants were female, the mean age of participants was 53.7 and average weight was 99.3 kg. Additionally, 11.1% of the sample identified as Aboriginal and/or Torres Strait Islander. Most participants lived in inner regional (30.8%) and outer regional (36.8%) areas and were in the lowest two socioeconomic status categories (63.4%). Participants completed an average of six of eight weekly sessions.

Eight-week follow-up assessments were obtained for 265 participants (64%). A comparison was undertaken of those who completed vs. those who did not complete the follow-up assessment (Table S2). Compared with non-completers, participants who completed the 8-week assessment were older (M = 55.0 ± 13.6 vs. M = 51.4 ± 14.3, *p* = 0.02), had a lower weight (M = 95.6 ± 22.1 vs. M = 105.9 ± 26.3, *p* < 0.001), smaller waist circumference (M = 111.5 ± 17.0 vs. M = 117.9 ± 16.8, *p* < 0.001), more 30 s chair stands (M = 11.2 ± 3.5 vs. M = 10.4 ± 3.6, *p* = 0.048), and completed more weekly sessions (M = 6.7 ± 1.5 vs. M = 4.0 ± 2.7, *p* < 0.001). Completion differed by remoteness (*p* = 0.003); rates of completion in major cities (100.0%) were similar to inner regional (57.7%), outer regional (68.0%), and remote SA (70.8%) but higher than very remote SA (33.3%). Completion also differed by socioeconomic status (*p* = 0.02); rates of completion in deciles 5–6 (55.1%) were lower than in deciles 3–4 (74.0%), while deciles 1–2 (59.8%), 3–4 (74.0%), 7–8 (73.5%), and 9–10 (57.1%) were similar. There were no differences in completion based on gender, Aboriginal and/or Torres Strait Islander status, health care card status, systolic blood pressure, diastolic blood pressure, walking, planned physical activity, incidental physical activity, total physical activity, sitting time, servings of fruit, servings of vegetables, 6 min walk test, or 30 s arm curl.

### 3.2. Program Effectiveness

At 8 weeks, statistically significant increases were shown in walking, planned physical activity, incidental physical activity, total physical activity, 30 s chair stand, 30 s arm curl, 6 min walk, fruit consumption and vegetable consumption. Statistically significant reductions were seen in sitting time, weight, waist circumference, and diastolic blood pressure. There were no changes in systolic blood pressure. Table 2 provides an overview of descriptive statistics and effectiveness results.

### 3.3. Satisfaction

Overall, 126 program participants (66% female) completed the satisfaction survey One hundred percent reported that they would recommend the program to family and friends and that the program was run at a convenient place. Most participants reported that the program was run at a convenient time (98%), that the quality of presentation was ‘excellent’ (89% vs. ‘good’ 10%, and ‘fair’ 0.8%), and that they were ‘confident’ or ‘very confident’ that they were able to make changes to their lifestyle as a result of the program (98%). Participants reported that the program raised their awareness of the health benefits of healthy eating and physical activity ‘a lot’ (90%), ‘a little’ (7%), or ‘not much’ (3%). Most participants reported that the program increased their healthy eating and physical activity skills by ‘a lot’ (71%) or ‘a little’ (26%) and many reported that the program prompted them to want to change their eating and physical activity habits ‘a lot’ (73%) or ‘a little’ (25%).

A total of 22 of the 30 stakeholders invited (73%) provided feedback; this included 10 program facilitators, 9 referrers and 3 practice/service managers. A minimum of one stakeholder from each region of delivery responded to the survey. One hundred percent of stakeholders reported that they were ‘very satisfied’ or ‘satisfied’ with the program and that they were ‘very likely’ or ‘likely’ to recommend the program to potential participants and colleagues/other health professionals. All respondents either ‘strongly agreed’ or ‘agreed’ that the program both met the needs of participants and was beneficial for people with chronic disease. Most (90%) reported the program is effective and appropriate for chronic disease management. The majority (82%) ‘strongly agreed’ or ‘agreed’ that the referral process was simple and easy, with the remaining ‘neither agreeing nor disagreeing’ (5%) or ‘disagreeing’ (14%). 95% ‘strongly agreed’ or ‘agreed’ that the program aligned with existing prevention and management programs within their organisation. A total of 60% reported (‘a great deal’ and ‘a lot’) that 2 h weekly group sessions for 8 weeks is sufficient for promoting positive behaviour change, with 40% reporting (‘a great deal’ and ‘a lot’) that this was sufficient for improving self-management of chronic disease risk factors. Ratings were lower regarding receiving adequate communication, with 40% of respondents reporting that they received adequate communication and updates on the progress of people they had referred. Among facilitators (*n* = 13), 85% reported they received adequate training to deliver HEAL^TM^ and 77% reported that the resources provided were ‘extremely’ or ‘very effective’, with the remaining reporting that they were ‘somewhat effective’ (23%).

## 4. Discussion

This study set out to determine the uptake, retention, effectiveness and acceptability of a group-based healthy lifestyle intervention delivered in rural and disadvantaged communities under “real-world” conditions. Overall, the results were positive, with strong referral to the program from a large number of health care providers. The program uptake rate was high amongst those referred. On average, participants completed six out of eight program sessions, and around half of participants completed the 8-week follow-up assessments, showing measurable improvements in most behavioural and physiological parameters measured. Participants’ feedback was highly favourable. Stakeholder feedback was also generally favourable, although referring clinicians wanted further communication regarding progress of the people they had referred into the program.

Results suggested that the HEAL^TM^ program led to measurable improvement in participants’ lifestyle behaviours. On average, program completers reported consuming two servings of fruit per day, which meant that by the program’s end, the average participant was meeting the recommended daily intake of fruit as per the Australian dietary guidelines [35]. Vegetable consumption increased substantially (0.8 increase in daily servings), though the average completer still fell short of healthy eating guidelines [35,36]. Self-reported physical activity increased by 140 min per week, representing a very large and clinically meaningful increase. It is important to acknowledge that these are self-reported changes, which are susceptible to social-desirability bias [37]. Previous research has highlighted that significant improvements in self-reported outcomes may not be reflected in significant changes when they are measured objectively [38].

Improvements seen in a variety of objectively measured health outcomes suggest that program participants did make meaningful changes to their lifestyle. In particular, participants, on average, lost approximately 1.4 kg. This degree of weight loss is comparable to, or perhaps slightly better than, one other study based on real-world delivery of the HEAL^TM^ intervention, in which completers lost, on average, 1.0 kg [23]. In contrast, a meta-analysis of the effects on 28 interventions modelling on the Diabetes Prevention Program found they led to 4 percent body weight loss at 12 months (vs. 1.4% loss at 8 weeks in our study) [22]. The comparability of these results is unclear, given their contrasting length of follow-up. The improvements in diastolic blood pressure, 6 min walk test and 30 s chair rise are all in line with improvements previously reported for the HEAL^TM^ intervention.

A particularly important finding from the current study was the high level of intervention uptake and acceptability. This is especially important when viewed in the light that the healthy lifestyle intervention was delivered in high-need communities that are typically hard to reach. Eleven percent of program participants were indigenous Australians (compared with 3.3% percent for the entire Australian population) [39] and SEIFA values indicate that the program was delivered in disadvantaged regions. This contrasts with many research-based health lifestyle programs, which typically reach white, relatively advantaged participants [40,41]. Whilst the program had good penetration in disadvantaged regions, loss to follow up was associated with SEIFA, highlighting the challenges of retaining socioeconomically disadvantaged participants across a prolonged period. Additionally, the current study continued that trend often seen in researcher-led programs, where they both attracted a large proportion of female participants [42].

The key strength of this study was that it evaluated a community-based physical activity and healthy eating program delivered under real-world conditions. Not only did the intervention reach a large number of underserved participants, but it was embedded within the existing health care system, with participants referred through other local health service providers. The reporting of the program’s results in the peer-reviewed literature demonstrates a clinician-instigated collaboration between health service providers and health researchers. Such collaborations are vital to support the reporting of real-world outcome data in the peer-reviewed literature [43]. At present, the largest body of peer-reviewed evidence regarding lifestyle interventions comes from researcher-led interventions, which often are discontinued at the end of the research project, and are not delivered under real-world conditions (e.g., participation is incentivised through financial payments, recruitment methods are not embedded in the health system, and participants are provided extensive support to complete the program and assessments).

Limitations must also be acknowledged. As is common for real-world intervention evaluations, a pre–post design was used with no control group. In addition, data relating to reasons for declining participation and attrition and drop-out were not captured Furthermore, physical activity and dietary outcomes were self-reported using simple instruments suited to a clinical setting, with modest validity relative to gold standard research measures. However, the changes in objectively measured health outcomes suggest that behaviour change was achieved. A further limitation was that blood pressure was measured using clinical-grade sphygmomanometers available at each site, but the brand and model were not recorded. However, given that the same sphygmomanometer was used for pre and post measures within participants, this should not have influenced the results, which focused on changes in blood pressure over time. Whilst these limitations might be considered major weaknesses for a traditional efficacy study, the health benefits of physical activity and healthy eating are well established, so the primary contribution of this study relates to implementation outcomes (e.g., uptake and acceptability). A key limitation was that the program was delivered and evaluated over a relatively short period due to funding constraints. Thus, the longer-term impacts are unknown. Ideally, real-world lifestyle programs should embed long-term follow-up assessment procedures to capture long-term impacts.

Overall, results suggest the HEAL^TM^ program was successfully delivered into these underserved rural communities, with strong uptake, reasonable intervention attendance, and excellent program acceptability. In the future, longer-term evidence and cost-effectiveness data would be valuable to support funding for ongoing programming and scale-up. Further work is needed to improve referral of, and program uptake, amongst men, who were under-represented in this study, and are characteristically reticent to partake in preventive health programs. The program may be improved by building in a communication mechanism by which participants’ progress is reported back to their referring health care providers.

## 5. Conclusions

In conclusion, this study evaluated the real-world uptake, retention, effectiveness and acceptability of a group-based healthy lifestyle intervention delivered in rural and underserved communities. The program achieved strong referral from clinicians, and uptake from participants. Around half of participants completed the 8-week program and follow-up assessments, with measurable improvements in behavioural and physiological outcomes. Future collaborative research between health service providers and researchers is warranted to establish the cost-effectiveness of the program and improve participation amongst men.

## Figures and Tables

**Table 1:** Participant characteristics at baseline (*n* = 414).

	*n*	Males (*n* = 102)	Females (*n* = 300)	All (*n* = 414) ^a^
Age (years), M (SD)	383	55.3 (12.55)	53.2 (14.4)	53.7 (13.9)
Aboriginal, *n* (%)	380	12 (12.2)	30 (10.6)	42 (11.1)
Health care card (yes), *n* (%)	362	53 (55.8)	113 (49.8)	186 (51.4)
Remoteness, *n* (%)	399			
Major Cities		3 (2.9)	5 (1.7)	8 (2.0)
Inner Regional		30 (29.4)	93 (31.5)	123 (30.8)
Outer Regional		47 (46.1)	99 (33.6)	147 (36.8)
Remote		18 (17.6)	87 (29.5)	106 (26.6)
Very Remote		4 (3.9)	11 (3.7)	15 (3.8)
SES decile, *n* (%)	399			
1–2		36 (35.3)	71 (24.1)	107 (26.8)
3–4		26 (25.5)	119 (40.3)	146 (36.6)
5–6		28 (27.5)	70 (23.7)	98 (24.6)
7–8		5 (4.9)	28 (9.5)	34 (8.5)
9–10		5 (6.9)	7 (2.4)	14 (3.5)
Completed sessions, M (SD)	290	6.2 (2.3)	6.1 (2.0)	6.1 (2.1)
Walking (min/week), M (SD)	394	91.8 (118.9)	71.6 (99.3)	75.7 (103.9)
Planned PA (min/week), M (SD)	397	66.7 (112.2)	56.1 (96.7)	59.1 (100.2)
Incidental PA (min/week), M (SD)	378	119.7 (170.9)	116.6 (170.5)	116.9 (169.0)
Total PA (min/week), M (SD)	358	277.0 (282.6)	230.2 (227.7)	240.7 (241.5)
Sitting time (h/day), M (SD)	400	6.7 (3.8)	5.8 (2.9)	6.03 (3.15)
Fruit (servings/day), M (SD)	409	1.5 (1.1)	1.6 (1.2)	1.6 (1.1)
Vegetables (servings/day), M (SD)	411	2.4 (1.3)	2.6 (1.4)	2.6 (1.4)
Weight (kg), M (SD)	413	109.9 (26.8)	95.6 (22.3)	99.3 (24.2)
Waist circumference (cm), M (SD)	404	120.4 (16.9)	111.5 (16.8)	113.8 (17.2)
BMI, M (SD)	412	35.8 (7.8)	36.6 (8.0)	36.4 (7.9)
Systolic BP (mmHg), M (SD)	384	139.8 (14.8)	133.3 (15.8)	135.1 (15.7)
Diastolic BP (mmHg), M (SD)	377	81.0 (10.0)	79.0 (10.4)	79.5 (10.3)
30 s chair stand (*n*), M (SD)	396	10.9 (3.8)	10.9 (3.5)	10.9 (3.5)
30 s arm curl (*n*), M (SD)	403	23.0 (9.5)	22.2 (9.5)	22.3 (9.4)
6 min walk (m), M (SD)	377	380.0 (127.6)	375.0 (127.8)	376.6 (130.2)

^a^ All is more than the sum of Male and Female, due to missing gender data. Note: SES = socioeconomic status, PA = physical activity, BP = blood pressure.

**Table 2:** Descriptive statistics and results from multilevel models examining the effectiveness of the HEAL^TM^ program.

	Baseline		8 Weeks		Estimated Difference
	*n*	M (SD)	*n*	M (SD)	B [95% CI]
Walking (min/week)	394	75.7 (103.9)	250	110.7 (116.7)	31.66 [20.77, 42.56] ***
Planned PA (min/week)	397	59.1 (100.2)	251	115.4 (99.9)	58.09 [45.88, 70.30] ***
Incidental PA (min/week)	378	116.9 (169.0)	239	157.2 (204.4)	46.17 [24.24, 68.11] ***
Total PA (min/week)	358	277.0 (282.6)	292	387.5 (277.3)	140.98 [112.87, 169.09] ***
Sitting time (h/day)	400	6.03 (3.15)	252	5.5 (2.7)	−0.36 [−0.59, −0.12] **
Fruit (servings/day)	409	1.6 (1.1)	255	2.0 (1.0)	0.38 [0.27, 0.48] ***
Vegetables (servings/day)	411	2.6 (1.4)	256	3.4 (1.4)	0.81 [0.66, 0.96] ***
Weight (kg)	413	99.27 (24.17)	263	94.45 (22.05)	−1.43 [−2.44, −0.41] **
Waist circumference (cm)	404	113.8 (17.2)	257	110.3 (17.8)	−1.61 [−2.50, −0.72] ***
Systolic BP (mmHg)	384	135.1 (15.7)	245	133.4 (15.4)	−1.63 [−3.42, 0.17]
Diastolic BP (mmHg)	377	79.5 (10.3)	239	78.3 (10.4)	−1.10 [−2.15, −0.05] *
30 s chair stand (*n*)	396	10.9 (3.5)	250	13.1 (4.9)	2.01 [1.61, 2.42] ***
30 s arm curl (*n*)	403	22.3 (9.4)	255	24.6 (10.0)	2.61 [2.03, 3.19] ***
6 min walk (m)	377	376.6 (130.2)	234	402.0 (146.9)	24.68 [12.70, 36.66] ***

* *p* < 0.05, ** *p* < 0.01, *** *p* < 0.001; Note: PA = physical activity, BP = blood pressure.

## Data Availability

This study is based on patient data. The patients did not provide consent for their individual data to be shared; therefore, the data are not available.

## Supplementary Materials

The following are available online at https://www.mdpi.com/article/10.3390/nu13114040/s1, Table S1: Comparison of individuals who accepted and did not accept the referral to HEAL^TM^, Table S2: Comparison of participants who completed the eight-week follow-up assessment and non-completers.
