# Supplementary material for: Effectiveness of a Lifestyle Modification Program Delivered under Real-World Conditions in a Rural Setting
Source: Nutrients. 2021 Nov 12;13(11):4040. doi: 10.3390/nu13114040 (PMC8620632; doi:10.3390/nu13114040)
Supplement: Supplementary file 1 [file nutrients-13-04040-s001.zip › nutrients-1414013-supplementary.pdf]

Table S1. Comparison of individuals who accepted and did not accept the referral to HEAL™.

	χ^2 or F	df	p
Age	3.05	1	.08
**Gender**	**6.11**	**1**	**.02**
Aboriginal and/or Torres Strait Islander	0.10	1	.83
**Healthcare card**	**7.33**	**1**	**.01**
Socioeconomic status	5.44	4	.25
**Remoteness**	**12.74**	**4**	**.01**

Note: Bold indicates significant.

Table S2. Comparison of participants who completed the eight-week follow-up assessment and non-completers.

	χ^2 or F	df	p
Age	**5.95**	**1**	**.02**
Gender	0.51	1	.48
Aboriginal and/or Torres Strait Islander	0.10	1	.87
Healthcare card	0.004	1	.95
Socioeconomic status	**12.1**	**4**	**.02**
Remoteness	**15.90**	**4**	**.003**
Completed sessions	**105.64**	**1**	**<.001**
Walking (min/week)	1.67	1	.20
Planned PA (min/week)	0.05	1	.82
Incidental PA (min/week)	1.60	1	.20
Total PA (min/week)	3.33	1	.07
Sitting time (hours/day)	2.00	1	.16
Fruit (serves/day)	1.94	1	.17
Vegetables (serves/day)	0.26	1	.61
Weight (kg)	**17.86**	**1**	**<.001**
Waist circumference	**13.24**	**1**	**<.001**
Systolic BP (mmHg)	2.28	1	.13
Diastolic BP (mmHg)	0.04	1	.84
30-sec chair stand (n)	**3.95**	**1**	**.48**
30-sec arm curl (n)	1.56	1	.22
6-min walk (m)	0.99	1	.32

Note: Bold indicates significant.
